# An Observational Investigation of Behavioral Contagion in Common Marmosets (*Callithrix jacchus*): Indications for Contagious Scent-Marking

**DOI:** 10.3389/fpsyg.2016.01190

**Published:** 2016-08-09

**Authors:** Jorg J. M. Massen, Vedrana Šlipogor, Andrew C. Gallup

**Affiliations:** ^1^Department of Cognitive Biology, University of ViennaVienna, Austria; ^2^Psychology Department, State University of New York at OneontaOneonta, NY, USA

**Keywords:** behavioral contagion, marmosets, yawning, scent-marking, yawn contagion, contagious yawning

## Abstract

Behavioral contagion is suggested to promote group coordination that may facilitate activity transitions, increased vigilance, and state matching. Apart from contagious yawning, however, very little attention has been given to this phenomenon, and studies on contagious yawning in primates have so far only focused on Old World monkeys and apes. Here we studied behavioral contagion in common marmosets, a species for which group coordination and vigilance are paramount. In particular, we investigated the contagiousness of yawning, stretching, scratching, tongue protrusion, gnawing, and scent-marking. We coded these behaviors from 14 adult marmosets, from two different social groups. During testing sessions, animals were separated into groups of four individuals for 20-min observation periods, across three distinct diurnal time points (morning, midday, and afternoon) to test for circadian patterns. We observed almost no yawning (0.12 yawns/h) and very little stretching behavior. For all other behaviors, which were more common, we found several temporal and inter-individual differences (i.e., sex, age, dominance status) predictive of these responses. Moreover, we found that gnawing and scent-marking, which almost always co-occurred as a fixed-action pattern, were highly temporally clustered within observation sessions. We discuss the relative absence of yawning in marmosets as well as the possible function of contagious scent-marking, and provide suggestions for future research into the proximate and ultimate functions of these behaviors in marmosets.

## Introduction

Contagious behavior can be defined as “behavior that is automatically triggered, or released, by the similar behavior of others” (Zentall, 2003). Similarly, response facilitation has been used to describe how the mere observation of a particular behavior by another increases the frequency of the observer performing the same action (Byrne, 1994). From a proximate perspective, hypotheses regarding the underlying causes of behavioral contagion range from mechanisms rooted in primitive forms of state matching and empathic processing (e.g., Joly-Mascheroni et al., 2008; Palagi et al., 2009; Campbell and de Waal, 2011) to more parsimonious explanations like non-conscious mimicry or just a short-term spread of a behavior in which a special stimulus “serves as a releaser to the unlearned behavior of others” (Zentall, 2001; Yoon and Tennie, 2010). Functionally, patterns of behavioral contagion have been suggested to promote group coordination that may aid in rest-activity transitions (Deputte, 1994), social cohesion (Conradt and Roper, 2000), vigilance (Miller et al., 2012a; Hare et al., 2014), and/or state matching (Osvath and Sima, 2014).

Perhaps one of the best examples of a contagious behavior is yawning. In humans, yawns can be elicited simply by thinking about yawning (Provine, 1986), and experimentally it has now been shown that seeing (Provine, 1986; Platek et al., 2003) or hearing (Massen et al., 2015) other people yawn increases the probability of yawning. Studies investigating the contagiousness of yawning in non-human animals have increased dramatically in the past decade. To date, contagious yawning has been reported for chimpanzees (Anderson et al., 2004; Campbell et al., 2009; Campbell and de Waal, 2011; Massen et al., 2012; Amici et al., 2014), bonobos (Demuru and Palagi, 2012; Palagi et al., 2014; but see Amici et al., 2014), gelada baboons (Palagi et al., 2009) domestic dogs in response to humans (Joly-Mascheroni et al., 2008; Silva et al., 2012; Madsen and Persson, 2013; Romero et al., 2013; but see Harr et al., 2009; O'Hara and Reeve, 2011; Buttner and Strasser, 2014), budgerigars (Miller et al., 2012b; Gallup et al., 2015), wolves (Romero et al., 2014), and a sub-line of high-frequency yawning rats (Moyaho et al., 2015). In contrast, experimental studies have failed to show contagious yawning in bonobos, orangutans, and gorillas to both human demonstrators and conspecifics (Amici et al., 2014), and in ring-tailed and ruffed lemurs (Reddy et al., 2016), dogs (Harr et al., 2009), and red-footed tortoises (Wilkinson et al., 2011) in response to conspecifics. Furthermore, a study on stump-tailed macaques (Paukner and Anderson, 2006) showed that yawning could be induced in this species by showing videos of yawning conspecifics, yet the co-occurring high frequencies of self-directed behaviors alongside the video presentation suggested that the enhanced yawning was likely due to stress rather than contagion, again emphasizing the difficulties in interpreting possible underlying mechanisms.

To date, all ape species as well as some Old World monkey species have been either experimentally tested or studied using observational techniques for their sensitivity to yawn contagion and various forms of behavioral response facilitation (Amici et al., 2014). A recent study on lemurs that did not find yawn contagion, suggests that either yawn contagion in haplorhine primates evolved after the lineage split from strepsirhines, or that it is ancient but has evolved to be more prevalent in haplorhines (Reddy et al., 2016). Little to nothing is known about the presence or absence of yawn contagion in New World monkeys, despite the potential insights such investigations could provide in tracing the phylogeny (i.e., the split between platyrrhines and catarrhines) of this behavioral response.

Other behaviors aside from yawning have also been described as contagious in humans, as well as in other species, such as laughter (humans: Provine, 2005), itch and associated scratching (humans: Holle et al., 2012; rhesus macaques: Feneran et al., 2013), stretching (budgerigars: Miller et al., 2012b), sniffing (humans: Arzi et al., 2014), play (ravens: Osvath and Sima, 2014), and “jump-yip” displays (prairie dogs: Hare et al., 2014). Nevertheless, in general the occurrence of behavioral contagion remains relatively understudied.

Here, we set out to investigate the contagious properties of several behaviors observed in common marmosets (*Callithrix jacchus*). To do so, we studied the temporal distribution of these behaviors in groups to identify patterns of non-random clustering or clumping, which is an indication of contagion (cf. Miller et al., 2012a,b). Moreover, we subsequently charted inter-behavior intervals to identify latencies in response following cues (i.e., priming behaviors) from adjacent conspecifics. Clustering of observed behavior can also be a result of demographic or circadian patterns, and therefore, we also analyzed the effects of sex, age, dominance status, and time of day on our behavioral variables. Moreover, such demographic and/or circadian patterns could potentially illuminate the function of these behaviors by improving our understanding of the naturalistic frequency of such responses. Nevertheless, in case of true contagion, we predict that even within a demographic- or temporal-category, these behaviors will be clustered.

Common marmosets are New World monkeys native to Brazil, which live in small family groups (Ferrari and Digby, 1996), and behavioral synchronization, group cohesion, and vigilance might be particularly relevant for them. Consequently, the study of behavioral contagion in this species seems appropriate. Marmosets are obligate cooperative breeders (Ferrari and Digby, 1996), and this particular breeding system has been suggested to require coordinated cooperation and keen attentiveness to others' behavior (Burkart and van Schaik, 2010). Moreover, because of their small size, they are rather vulnerable to predation (Grzimek, 2003), and group cohesion and vigilance are thus paramount. Indeed, common marmosets have been shown to exhibit conformity with regard to socially learned foraging techniques (Gunhold et al., 2014) and with regard to certain personality traits (Koski and Burkart, 2015; Šlipogor et al., 2016), and these “group-personalities” have been suggested to enhance cooperation and group cohesion. Social contagion of agonism and affiliation has already been shown between groups of marmosets (Watson and Caldwell, 2010), and can even be experimentally induced (Watson et al., 2014). These studies suggest a neighbor effect in marmosets, but within-group contagion remains unstudied in this species. Nonetheless, we predicted that marmosets would also be susceptible to behavioral contagion within their own group.

In particular, based on existing research we studied the temporal distribution of yawning, stretching, and scratching in 14 captive common marmosets from two different family groups. In addition, we also investigated the temporal distribution of three species-specific behaviors not yet studied for their potentially contagious properties; i.e., tongue protrusion, scent-marking, and gnawing. We included tongue protrusions because of its relative high frequency, and because in their original ethogram Stevenson and Poole (1976) mention that in common marmosets yawns are accompanied by the protrusion of the tongue. Nevertheless, we considered, and therefore analyzed, both behaviors as separate behaviors. We included scent-marking because for two ‘closely’ related tamarin species this behavior has been reported to be collective; i.e., “two or more individuals marked the same site sequentially or simultaneously” (Heymann, 2001), and thus seems like an apt candidate for a contagious behavior. Finally, we examined gnawing (cf. gouging: Lazaro-Perea et al., 1999) since from personal observations it has been noted in high frequency and therefore may be socially elicited.

## Methods

### Subjects and housing

Subjects were 14 adult common marmosets (*C. jacchus*; seven males, seven females) of two different family groups (named Kiri and Pooh) ranging in age between six and 13 years. Each group was comprised of the dominant breeding pair and five or six adult offspring (in one group one adult individual never participated, leading to seven test subjects per group). All individuals were born in captivity and housed at the Animal Care Facilities of the Department of Cognitive Biology, University of Vienna, Austria. All animals had access to both an indoor (250 × 250 × 250 cm) and outdoor (250 × 250 × 250 cm) enclosure, as well as to an experimental compartment (150 × 40 × 110 cm). Note that each group had its own experimental compartment, and that in these experimental compartments as well as in the in- and out-door home enclosures there was no visual access to any other group. Temperature (24–26°C), humidity (40–60%), and dark:light cycle (12:12 h) were kept as stable as possible within the indoor facilities. Every day at roughly 12:00 h the animals were fed a varied diet containing different fruits, vegetables, grains, milk products, pellets, marmoset jelly, protein and vitamin supplements, and insects. Water was available *ad libitum* in every compartment.

### Ethics

Housing conditions were in accordance with Austrian legislation and with the European Association of Zoos and Aquaria (EAZA) husbandry guidelines for Callitrichidae. The study complied with the International Primatological Society (IPS) guidelines for the use of non-human primates in research, and due to its observational nature, was exempted from overview by Austrian law (Austrian Animal Experiments Act § 2, Federal Law Gazette 2012).

### Procedure

At the start of each observational session four individuals from one family group entered the experimental compartment. We observed the animals in smaller experimental compartments as it allowed us to detect the subtle behaviors of the animals, which would not be possible in their home-cages. Additionally, the smaller compartment assured that the individuals in there were also able to easily observe and sense the behaviors from each other, a prerequisite for contagion. All individuals were used to regularly enter the experimental compartments, and already participated in experiments conducted using these experimental cages (e.g., Gunhold et al., 2015). Consequently, the animals showed no signs of stress whenever they were in these compartments. Additionally, to be able to reliably observe these subtle behaviors, group size was held constant at four. Due to the fact that participation of individuals was on a voluntary basis, however, group composition in the testing compartments was variable. However, most of our subjects (11 out of 14) participated at least once in each of the three diurnal distinct periods [see below and see Supplemental Information (SI) for details on group composition per session]. After a short acclimation period of ~5 min when the individuals steadily decreased their level of excitement; i.e., locomotion, abrupt jumps, etc., we started observation sessions lasting 20 min. To avoid over- or under-representation of behaviors due to circadian rhythms, we conducted observation sessions during three distinct diurnal periods: In the morning (starting time ranging between ~8:30 and 10:15 h), around midday (starting time ranging between ~12:00 and 13:00 h), and during the afternoon (starting time ranging between ~15:00 and 16:00 h). With one group (Kiri) we conducted seven observation sessions per each time period, whereas with the second group (Pooh) we conducted a total of four morning sessions, seven midday sessions and six afternoon sessions. Due to conflicts among the test subjects, we had to break up three sessions before the end of the 20 min. In these cases all data prior to the conflict was included in the analyses where applicable. All sessions were simultaneously videotaped from two different angles (JVC Everio).

### Analyses

All videos were coded by VŠ using Solomon coder (Péter, 2011). The videos were coded for the following behaviors: yawning, stretching, scratching, tongue protrusions, gnawing/gouging on wood/structures, and scent-marking (cf. ethogram of Stevenson and Poole, 1976; http://www.marmosetcare.com/). All behaviors were coded as events and had a duration < 2 s. A naïve research assistant recoded 10.5% of the videos and overall agreement was near perfect (Cronbach's alpha = 0.977, *p* < 0.001).

To test for the effects of sex, age, dominance status, and time of day on our behavioral variables, we ran models on individual behavioral frequencies for each response per observation session. As these behavioral variables were not normally distributed we chose to use Generalized Linear Mixed Models (GLMMs) with an identity link option as this can be used with any distribution of the target (apart from a multinomial; IBM, 2015), and additionally GLMM's allowed us to reflect the nested structure of our data. In these GLMMs the behavioral variable of interest was entered as the target, and age (years), sex, dominance status (dominant breeding pair vs. adult offspring/helper), and time of day (morning, midday, or afternoon) were entered as fixed factors. To avoid pseudo-replications we structured our data to be nested within each individual and individuals were nested in their corresponding group. In addition, we included both group and individual identity as random factors in the GLMMs. We ran models including all main factors and reduced models and selected the best fitting models based on comparisons of corrected Akaike Information Criteria (cAIC), which correct for finite sample sizes (Hurvich and Tsai, 1993). Model assumptions were examined by investigation of the Quantile-Quantile (Q-Q) plots comparing the distribution of the residuals of each model with a normal distribution. Apart from some outliers, the residuals of our models showed to be relatively normally distributed (see SI). When appropriate, we calculated *post-hoc* comparisons using Wilcoxon signed ranks tests based on individual means per category of comparison.

Analyses of contagion followed the same procedures performed by Miller et al. (2012b) for assessing contagious behavior in budgerigars. Contagion would be represented by behaviors being clustered in time rather than randomly distributed, and this can be detected by examining whether behavioral runs are more common than would be expected by chance. The times between adjacent matched behaviors were calculated and frequencies of occurrence were binned into 20-s intervals. To identify significantly non-random distributions of intervals, separate runs tests were performed across all observational sessions (38 per behavior; 228 total). Therefore each 20-min session was broken into sixty 20-s bins and each behavioral frequency was calculated separately for each bin. A run was composed of consecutive bins (two or more) identified as either having at least one of the particular behaviors (1), or not having the said behavior (0). There was no distinction between bins with one vs. multiple behaviors, e.g., a 20-s bin with four said behaviors was treated the same as a 20-s bin with just one behavior. The runs test compares the observed number of runs to what would be expected given the behavioral frequency across the session. The generated *Z*-score is normally distributed, with negative values indicating a greater degree of temporal clustering or clumping (i.e., patterns of both consecutive bins with and without a particular behavior). Alternatively, positive *Z*-scores indicate a greater than expected dispersion of behavior across the group. These tests fail to yield *Z*-scores in cases whereby only a single run (i.e., all 1s or all 0s) occurs throughout the testing session. Thereby testing sessions where behaviors were overabundant or completely absent were removed from this analysis. This resulted in the removal of 35 sessions for yawning, 20 sessions for stretching, and one session for gnawing due to complete absence, while only one session for scent-marking was removed due to overabundance. Furthermore, any trials that had less than 10 said behaviors were checked for individual bin repeats, and sessions were removed from the analysis in cases where consecutive bins resulted from one individual displaying the same behavior multiple times (i.e., creating a false signal of contagion). This included a total of 15 behavioral sessions, spread across yawning (1), stretching (2), scratching (8), and tongue protrusion (4).

We then performed a combined probability test, as described by Sokal and Rohlf (1995; pp. 778–782), to determine the probability of non-random clumping across all test sessions for each behavior. The combined probability test was therefore used to evaluate a common null hypothesis that was independently tested by each runs test, but in this case indicates whether there was an overall significant bias in one direction (i.e., temporal clumping or dispersion) across all observational sessions when taking into account the probability values from each runs test. In addition, we could assess whether certain behaviors were more contagious between groups and across different times of day. Importantly, Pearson correlations revealed no relationship between the number of times a particular behavior was performed and the degree of temporal clustering across group members. In other words, simply because a behavior is more frequent across the group does not mean it is considered more contagious.

Lastly, the inter-behavior interval was assessed for behaviors appearing to be contagious through the aforementioned analyses. In particular, the latency for response, as measured within 20-s bins ranging from 20-s to 120-s or greater, was calculated separately for each behavioral expression by identifying the timing of the most recently performed matched behavior from a separate group member in the experimental cage. If contagious, one would expect a higher frequency of closely spaced behaviors (20–40 s) followed by longer intervals until the occurrence of a new, first behavior (priming behaviors; Miller et al., 2012b). It is noteworthy that this approach has advantages over simply comparing the behavioral frequencies per animal with and without social cues at a fixed latency (e.g., Palagi et al., 2009) because it provides the same information (frequency of response with and without cues) while also exploring the potential bimodal nature of the temporal distribution predicted by contagion.

Analyses were calculated using SPSS (version 23.0, IBM, Armonk, USA). All reported *P*-values are two-tailed and alpha was set to 0.05.

## Results

### Descriptive analyses

#### (For graphical representation of significant effects, please see SI)

The videos confirmed that yawning and tongue protrusion are different behaviors in marmosets; i.e., whereas tongue protrusions are performed with a relatively relaxed open mouth and open eyes, yawns are characterized by a wide opening/stretching of the mouth and the closing of the eyes, and no protrusion of the tongue (cf. Barbizet, 1958; Provine, 1986; and see Supplementary Video 1 for an example of a marmoset that clearly shows the difference). However, from 49.87 focal hours of observation on 14 individuals (x ± SE = 3.56 ± 0.64 h per individual) we observed a total of only six yawns by two individuals, one of each group; i.e., Kiri (of the Kiri group) yawned four times in one session only, and from Pooh (of the Pooh group) a single yawn was recorded in two separate sessions. Due to the scarcity of this behavior, we rendered any additional analyses uninformative.

Stretching was also relatively rare as we observed it only 42 times, although 12 out of the 14 individuals showed the behavior at least once. None of our descriptive variables; i.e., age, sex, dominance, or time of day, could explain the frequency of stretching per individual, since the best fitting model was the null model (i.e., intercept only).

On average the marmosets scratched themselves 0.11 ± SE 0.02 times/min. The model that best explained the variation revealed that scratching rates increase slightly, but significantly, with the age of the marmosets [β = 0.027, *F*_(1, 149)_ = 4.278, *p* = 0.04], and that dominant breeders scratched significantly less than helpers [β = − 0.142, *F*_(1, 149)_ = 5.082, *p* = 0.026].

The marmosets protruded their tongue on average 0.26 ± SE 0.02 times/min. Similar to scratching, the model that best explained the variation revealed that tongue protrusion rates increase slightly, but significantly, with the age of the marmosets [β = 0.057, *F*_(1, 147)_ = 4.734, *p* = 0.031], and that dominant breeders protruded their tongue significantly less than helpers [β = − 0.278, *F*_(1, 147)_ = 5.054, *p* = 0.026]. Additionally, the best fitting model regarding tongue protrusion rates revealed an effect of the time of day [*F*_(1, 147)_ = 3.463, *p* = 0.034], however, *post-hoc* comparisons did not find any significant differences between morning, midday, and afternoon (Wilcoxon signed ranks tests: morning vs. midday: *T*^+^ = 54, *n* = 12, *p* = 0.239; morning vs. afternoon: *T*^+^ = 21, *n* = 11, *p* = 0.286; midday vs. afternoon: *T*^+^ = 16, *n* = 11, *p* = 0.131).

**Figure 1:**
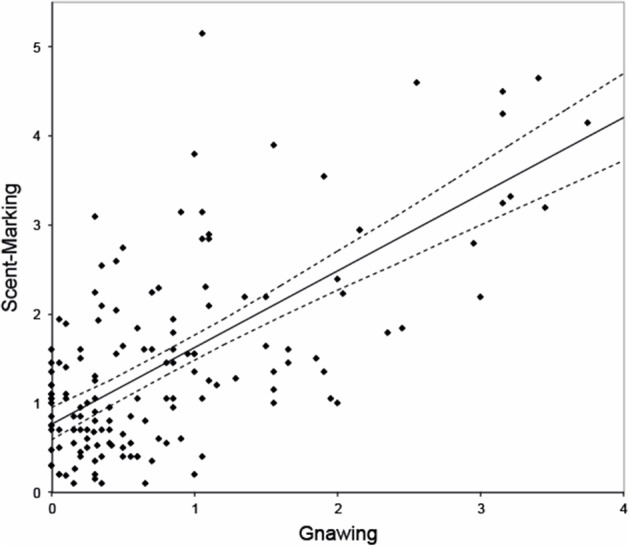
**Relationship between gnawing (frequency per animal per session) and scent-marking (frequency per animal per session)**. Dashed lines represent 95% confidence intervals. Note that the regression line here only serves as a graphical representation, though as it is based on an overall effect with multiple data-points per monkey it does not reflect the reported model.

Gnawing was observed on average 0.75 ± SE 0.18 times/min. The model that best explained the variation in this response revealed that females gnawed significantly less than males [β = −0.844, *F*_(1, 149)_ = 9.049, *p* = 0.003], and that dominant breeders tended to gnaw less than helpers, albeit not significantly [β = −0.583, *F*_(1, 149)_ = 3.634, *p* = 0.059]. During the observations, however, we noticed that gnawing frequently co-occurred with and preceded scent-marking. *Post-hoc* analysis confirmed a strong and significant relationship between both behaviors [β = 0.829, *F*_(1, 150)_ = 70.482, *p* < 0.001, Figure 1) in which gnawing positively predicts scent-marking.

Finally, regarding scent-marking, we observed very high rates of this behavior; i.e., the marmosets scent marked on average 1.37 ± SE 0.19 times/min. The model that best explained variation in this response revealed that females scent-marked significantly less than males [β = −0.908, *F*_(1, 146)_ = 7.596, *p* = 0.007], with a significant effect of time of day [*F*_(1, 146)_ = 3.148, *p* = 0.046]. *Post-hoc* analyses revealed that the marmosets scent-mark significantly less during the afternoon than during the morning (Wilcoxon signed ranks tests: morning vs. midday: *T*^+^ = 30, *n* = 12, *p* = 0.480; morning vs. afternoon: *T*^+^ = 11, *n* = 11, *p* = 0.050; midday vs. afternoon: *T*^+^ = 18, *n* = 11, *p* = 0.182). In addition, the best fitting model showed a tendency for the rate of scent-marking to increase with age [β = 0.251, *F*_(1, 146)_ = 3.661, *p* = 0.058], and revealed that dominant breeders tended to scent-mark less than helpers [β = −1.207, *F*_(1, 146)_ = 3.587, *p* = 0.060], albeit that both effects were not significant.

### Analyses of contagion

**Figure 2:**
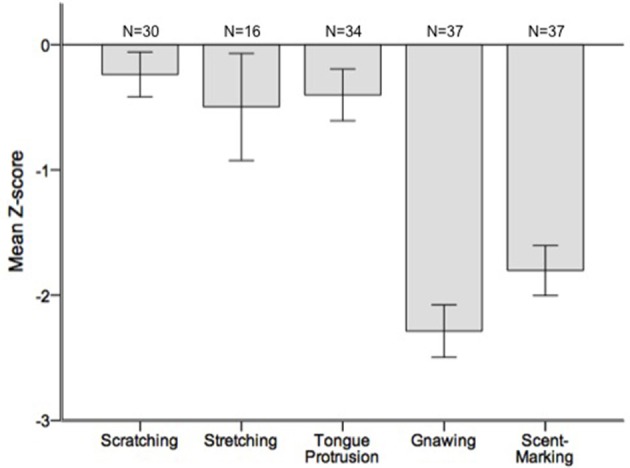
**Mean ± SEM Z-scores from the runs tests performed for each of the testing sessions**. The number of testing sessions included in the analysis is indicated above for each behavior.

**Figure 3:**
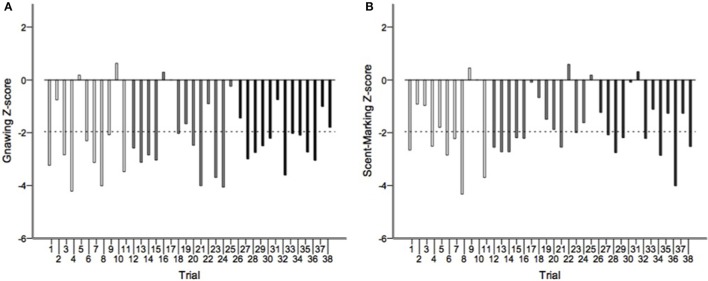
**The distribution of Z-scores from the runs test analyses for (A) gnawing and (B) scent-marking across all morning (white), midday (gray), and afternoon (black) testing sessions**. The dotted line indicates the threshold for significant temporal clumping within a session (*Z* < −1.960, *P* < 0.05). This was breached 26 times for gnawing, and 19 times for scent-marking.

**Figure 4:**
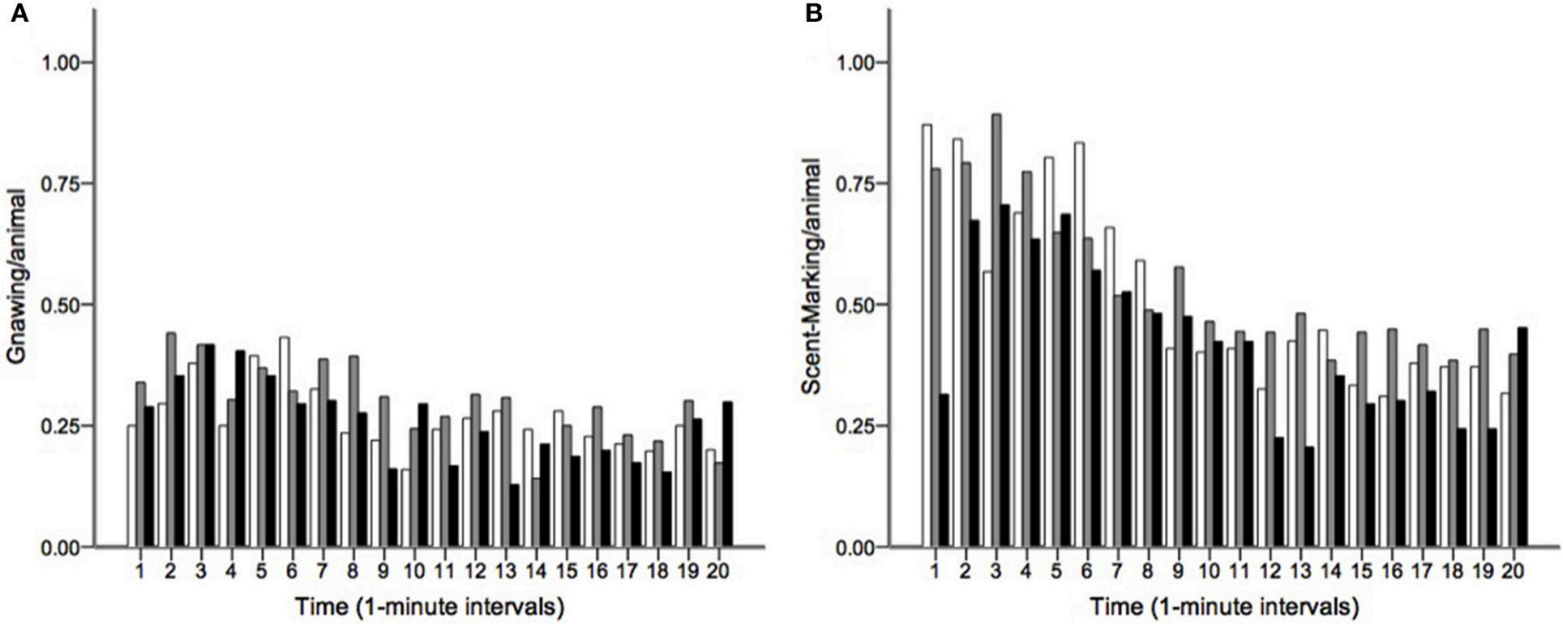
**The distribution of (A) gnawing and (B) scent-marking across the 20-min morning (white), midday (gray), and afternoon (black) testing sessions**. The mean number of gnaws and scent-marks per animal are shown at 1-min intervals, indicating that both behaviors occurred at greater frequency at the beginning of the trials.

Runs tests revealed significant temporal clumping (*Z* < −1.960, *p* < 0.05) in only three sessions for scratching, two sessions for stretching, and two sessions for tongue protrusion (see SI), while this non-random distribution was found in 26 sessions for gnawing and 19 sessions for scent-marking. None of the testing sessions revealed significant dispersal of any of the behaviors. Figure 2 depicts the average *Z*-scores for each of the behaviors. The combined probability tests confirmed that gnawing and scent-marking were non-randomly clustered in time across the testing sessions (gnawing: *X*${}_{\left(74\right)}^{2}$ = 305.392, *p* < 0.001; scent-marking: *X*${}_{\left(74\right)}^{2}$ = 228.608, *p* < 0.001; see Figure 3), while no other behaviors were significant. Figure 4 shows the relative distribution in spacing for gnawing and scent-marking across the testing sessions, indicating slightly higher frequencies (particularly for scent-marking) at the beginning of the observational periods for each diurnal time point. We found no differences in the degree of clumping for any of the behaviors between the two groups (*p* > 0.05) or across the three time points of the day (*p* > 0.05).

**Figure 5:**
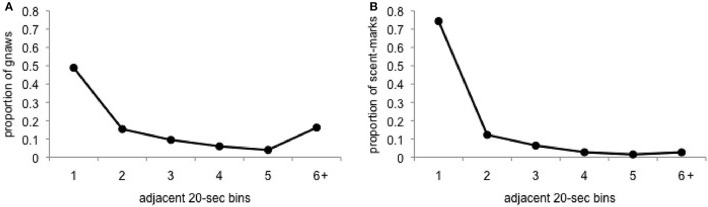
**Proportion of (A) gnaws and (B) scent-marks that occurred within 20-s bins, ranging from 20 to 120-s or greater, of the most recently performed matched behavior from a separate group member**.

Based on the outcome above, we then calculated the inter-behavior intervals for gnawing and scent-marking to further explore the potentially contagious nature of these behaviors. Consistent with the predictions of contagion, the majority of gnawing behavior occurs within 20–40-s of an adjacent animal's gnawing or after a long period of time without gnawing (>120-s). A similar, although much less bimodal, pattern is observed for scent-marking (Figure 5). In this case, while the majority of scent-marks were immediately preceded by the matched behavior in an adjacent group member, fewer instances of scent-marking were observed following a gap in response of at least 120-s.

## Discussion

In this study we investigated the temporal distribution of yawning, stretching, scratching, tongue protrusion, gnawing, and scent-marking in common marmosets. Notably, we observed hardly any yawning events. Similarly, stretching was relatively rare among the marmosets. Regarding all other behaviors, we found several sex, age, breeder-helper, and temporal differences in frequencies. Finally, we showed that gnawing and scent-marking were significantly non-randomly distributed and temporally clustered, suggesting these behaviors are contagious. Further analyses of inter-behavior intervals support this interpretation, though experimental tests are needed to confirm these findings.

Due to its relative absence, we could not investigate whether yawning is contagious in marmosets. However, from the few yawns that were recorded, we are able to show that yawning and tongue protrusion are different behaviors. Similar to yawning, the rarity of stretching may have also occluded its contagiousness, which has been reported in budgerigars (Miller et al., 2012b). However, the finding that spontaneous yawning is so uncommon among marmosets is noteworthy, because yawning is rather ubiquitous across vertebrates (Craemer, 1924; Luttenberger, 1975; Baenninger, 1987; Gallup et al., 2009) and, such low frequency yawning is relatively rare in primates (cf. Palagi et al., 2009, 2014; Zannella et al., 2015; the control conditions of Anderson et al., 2004; Paukner and Anderson, 2006; but see Reddy et al., 2016).

Functionally, recent studies have gathered support for a brain cooling function to yawning (Gallup and Gallup, 2007, 2008; Shoup-Knox et al., 2010; Gallup and Eldakar, 2013; Massen et al., 2014; Eldakar et al., 2015), and in humans frequencies of yawning vary non-linearly across ambient temperatures (Gallup and Eldakar, 2011; Massen et al., 2014; Eldakar et al., 2015), i.e., being most common within intermediate temperatures at or slightly above a thermal neutral zone and decreasing in a predicted fashion at temperature extremes. At our facilities the ambient temperature for the marmosets is kept constant at about 24–26°C, and yawning could still have a brain cooling function for marmosets, which have a core-body temperature of about 40°C (Cilia et al., 1998). Nevertheless, it has been suggested that yawning might not be as highly involved in behavioral thermoregulation across all primates (Gallup, 2010). In marmosets, alternative thermoregulatory behaviors like tongue protrusion may suffice. In capuchins, for example, it has been shown that frequencies of tongue protrusions increase with heat stress (Campos and Fedigan, 2009). However, yawn frequencies follow the same thermal patterns as tongue protrusions in capuchins (Campos and Fedigan, 2009), suggesting that yawning has an additional thermoregulatory function (Gallup, 2010). Since the geographical distributions and climatic conditions of common marmosets and capuchin monkeys overlap, it remains unclear why the marmosets hardly yawn whereas the capuchins do. One conspicuous anatomical difference between the two species is the presence of ear tufts in marmosets. We speculate that the ear tufts provide effective heat dissipation from the skull, therefore reducing the frequency of behavioral brain cooling mechanisms. Consistent with this interpretation, thermoregulatory functions have been proposed for the manes of lions (West and Packer, 2002), hairs on Saharan silver ants (Shi et al., 2015), and the ear tufts of bearded vultures (Margalida et al., 2008). Nevertheless, it is also possible that the low frequency yawning observed here is a result of the particular compartmental conditions and thus further research is needed to test this hypothesis.

In addition to a potential involvement in thermoregulation, there may also be alternative physiological mechanisms that trigger tongue protrusion since we noticed significant age and dominant status effects; i.e., with rates of tongue protrusion decreasing with increasing age, and with dominant breeders showing less tongue protrusions than subordinate helpers. As we found similar inter-individual patterns with regard to scratching rates, we suggest that stress and/or anxiety, which has been linked to self-directed behaviors like scratching in primates (Schino et al., 1996), may be a possible candidate. In contrast to baboons (Gesquiere et al., 2011), however, among marmosets there is no effect of dominance on stress, and, if anything, dominant breeders show higher stress levels than subordinates (Sapolsky, 2005). Moreover, the effects of aging on stress in non-human primates are thus far inconclusive (Goncharova, 2013). Furthermore, although we could show that yawning and tongue protrusion are different behaviors, in contrast to what has been suggested by Stevenson and Poole (1976), if we would consider them as equal or parallel behaviors, we did not find any evidence for contagion among marmosets.

Regarding gnawing, we identified that this behavior very often co-occurred with scent-marking, suggesting some sort of fixed-action pattern where the monkeys first gnaw on a piece of wood and then put a scent-mark in the place where they just gnawed. These results correspond to patterns in the wild showing that in 37.4% of all scent-marks, marmosets gouged the site prior to marking (Lazaro-Perea et al., 1999). The gnawing/gouging of the surface before leaving a mark is suggested to enhance the deposition and adherence of the scent-mark (Stevenson and Rylands, 1988), and may provide chemosensory information about the marking site via oral and nasal cavities (Epple, 1986). Analyses indicated that gnawing was non-randomly clustered or clumped in time across the group, which would be expected for a contagious response. Subsequent inter-gnaw intervals confirmed that the majority of gnawing behaviors immediately followed (20–40-s) the matched action in an adjacent group member. There was also a large proportion of gnawing behavior that was separated by at least 120-s from previous instances of gnawing. Together, this bimodal pattern supports the view that gnawing is at least in part socially influenced. However, since scent-marking is the more functional and most frequent of both behaviors, this is what we focus on for our discussion.

For scent-marking, the distribution varied across the day with marmosets showing a particularly high frequency of this behavior in the morning. This contrasts to a study of marmosets in their home enclosures (Nogueira et al., 2001), which reports higher frequencies of scent-marking in the afternoon. Marmosets have been shown to scent-mark particularly often when exploring new places (Epple, 1972; Stevenson and Poole, 1976), and for our observation sessions the marmosets came into separate experimental compartments. However, if it is just the novelty of the environment (note that the experimental compartments were actually not novel), one would expect a decrease of this behavior over the course of this study, a trend we did not observe. Alternatively, the activation for this behavior may be through olfactory cues and the cleaning of the experimental compartments immediately after each experiment may have triggered the scent-marking each time anew. Since these scents are very intense and persistent, however, some olfactory cues may remain after cleaning, and this would then explain why the frequencies of scent-marking were lower during the afternoon than in the morning. Additional *post-hoc* analyses support this hypothesis, revealing that there was only a difference between morning and afternoon if the afternoon session was preceded by another session of the same group (in the same experimental compartment) on the same day, be it morning or midday (*T*^+^ = 48, *n* = 10, *p* = 0.037), and not when the afternoon session was the only session of that day (*T*^+^ = 19, *n* = 8, *p* = 0.889). This suggests that in our setup scent-marking was predominantly used with regard to territorial defense. In general, there are three hypotheses about the functionality of scent-marking; (a) territory defense, (b) social status advertisement, and (c) reproductive status advertisement (Brown and Macdonald, 1985). The latter would predict more scent-marking of females than of males (Lazaro-Perea et al., 1999), yet we found the opposite pattern. Similarly, if scent-marking was used for social status advertisement, the prediction would be that dominant breeders scent-mark more than subordinates (Lazaro-Perea et al., 1999), yet our results showed a tendency for subordinates to be the predominant scent-markers. If the scent-marking in our setup indeed was mostly used for territory defense, the question why males and subordinates scent-mark more than females and dominants respectively remains unanswered. It may be that in this cooperative breeding species there is some sort of task specialization with regard to territory defense, much like, for example, within cooperatively breeding cichlids (Bruintjes and Taborsky, 2011). This may also explain the positive trend with regard to age, suggesting that younger marmosets perform other tasks. Alternatively, the trend with age may reflect a learning effect. Future experimental research is needed in order to test these potential explanations.

Similar to gnawing, scent-marking was also shown to be temporarily clustered, which is indicative of contagion; i.e., individuals were much more likely to scent-mark when another individual scent-marked within the preceding 20 s, in comparison to a random point in time. Inter-behavior intervals show that the majority of scent-marking was indeed immediately preceded (20–40-s) by scent-marking performed by an adjacent group member. But unlike gnawing, fewer scent-marking behaviors were separated by long intervals. This discrepancy makes sense, however, if in fact the overall fixed-action pattern is contagious since gnawing is the first act in the process, and thus a likely cue for contagion. Furthermore, the much higher frequency of scent-marking reduces the overall likelihood for there to be many instances separated by long bouts of time. Importantly, however, there was no correlation between behavioral frequencies and measures of contagion within testing sessions, and *post-hoc* analyses excluding the first sessions of a day (i.e., high frequency sessions, see above) still confirmed a temporal clustering for both gnawing and scent-marking (gnawing: *X*${}_{\left(24\right)}^{2}$ = 97.332, *p* < 0.001; scent-marking: *X*${}_{\left(26\right)}^{2}$ = 68.902, *p* < 0.001). That said, since over 75% of all scent-marking behavior occurred within 20-s of matched scent-marking from an adjacent animal, it could be that the smell of a scent-mark from a conspecific activates mechanisms leading to scent-marking in an individual. However, in a territorial defense context this would only be functional when that scent-mark was from someone of a different group, whereas the individuals in our testing sessions included only group members, and previous research has demonstrated that marmosets can discriminate familiar from unfamiliar conspecifics by their scent-mark (Smith et al., 1997). Nevertheless, coordination through contagion may enhance the signal on a group level. With regard to vigilance, chemosensory signals are particularly apt since they are distributed in a fast manner, and are efficient during night and day (Lübke and Pause, 2015), and thus contagion could serve to enhance a rapid spread of information through a social group. It remains unknown from the current study whether this is a visual or a truly chemosensory cue. Therefore, further experimental research (cf. Watson et al., 2014) is needed to identify the mechanisms involved in the suggested social transmission.

In summary, through semi-naturalistic observations of captive common marmosets, we provide the first data on the relative temporal distributions and various social effects of six distinct behaviors. In doing so, we reveal that yawning is extremely rare in this species, and to a lesser extent stretching is uncommon as well. Alternatively, scratching, tongue protrusion, gnawing, and scent-marking all occur at progressively greater frequency and appear to have social components, witnessed by various age, sex, and status effects. Lastly, we show indications that both gnawing and scent-marking, which appear in tandem and may contribute to a similar function, are likely to be contagious in this species. Overall, we believe these findings provide a strong foundation for future experimental research investigating the causal effects of exposure to conspecifics performing these behaviors, as well as the proximate mechanisms and ultimate functions associated with these effects in marmosets and other primates.

## Author contributions

JM and AG designed the study. JM and VS conducted the study and coded the videos. JM and AG analyzed the data. JM, VS, and AG wrote the paper.

### Conflict of interest statement

The authors declare that the research was conducted in the absence of any commercial or financial relationships that could be construed as a potential conflict of interest.
