# Supplementary material for: An Observational Investigation of Behavioral Contagion in Common Marmosets (Callithrix jacchus): Indications for Contagious Scent-Marking
Source: Front Psychol. 2016 Aug 9;7:1190. doi: 10.3389/fpsyg.2016.01190 (PMC4980586; doi:10.3389/fpsyg.2016.01190)
Supplement: Supplementary file 2 [file DataSheet1.DOCX]

Electronic Supplementary Materials for:

**An investigation of behavioural contagion in common marmosets (*Callithrix jacchus*): an observational study.**

Jorg J.M. Massen, Vedrana Šlipogor and Andrew C. Gallup

**Table S1.** Session number (#), data and time of day (M = Morning, N = Noon, A = Afternoon), and presence of individual marmosets per group.

|  |  |  | Kiri-group | | | | | | |
| --- | --- | --- | --- | --- | --- | --- | --- | --- | --- |
| # | Date | Time | Kiri | Veli | Mink | Jack | Nemo | Sparrow | Zaphod |
| 1 | 9/11/12 | N | x | x | x | x |  |  |  |
| 2 | 13/11/12 | M | x | x | x | x |  |  |  |
| 3 | 13/11/12 | A | x | x | x | x |  |  |  |
| 4 | 16/11/12 | M | x | x | x | x |  |  |  |
| 5 | 16/11/12 | A | x | x | x | x |  |  |  |
| 6 | 20/11/12 | M | x |  | x | x | x |  |  |
| 7 | 20/11/12 | N |  | x |  | x | x | x |  |
| 8 | 23/11/12 | N | x | x | x | x |  |  |  |
| 9 | 27/11/12 | A | x |  | x | x |  |  | x |
| 10 | 28/11/12 | N | x | x |  | x |  |  | x |
| 11 | 28/11/12 | A | x |  | x | x | x |  |  |
| 12 | 30/11/12 | N | x | x | x | x |  |  |  |
| 13 | 30/11/12 | A |  | x | x | x | x |  |  |
| 14 | 7/12/12 | M | x | x | x | x |  |  |  |
| 15 | 7/12/12 | A | x | x | x | x |  |  |  |
| 16 | 18/1/13 | M | x | x |  | x |  |  | x |
| 17 | 18/1/13 | N | x | x |  | x |  |  | x |
| 18 | 18/1/13 | A | x | x | x | x |  |  |  |
| 19 | 22/1/13 | M | x |  | x | x | x |  |  |
| 20 | 23/1/13 | N | x | x | x | x |  |  |  |
| 21 | 28/1/13 | M | x | x |  | x |  |  | x |
|  |  |  |  |  |  |  |  |  |  |
|  |  |  | Pooh-group | | | | | | |
| # | Date | Time | Pooh | Messina | Locri | Augustina | Pandu | Fimo | Yara |
| 1 | 9/11/12 | N | x | x | x | x |  |  |  |
| 2 | 9/11/12 | A | x | x | x |  | x |  |  |
| 3 | 13/11/12 | N | x |  | x | x | x |  |  |
| 4 | 16/11/12 | M | x | x | x |  | x |  |  |
| 5 | 16/11/12 | A | x | x | x | x |  |  |  |
| 6 | 20/11/12 | M | x | x | x |  | x |  |  |
| 7 | 20/11/12 | A | x |  | x |  | x | x |  |
| 8 | 23/11/12 | N | x | x | x |  | x |  |  |
| 9 | 23/11/12 | A | x |  | x |  | x | x |  |
| 10 | 27/11/12 | N | x | x | x |  | x |  |  |
| 11 | 28/11/12 | A | x | x | x |  | x |  |  |
| 12 | 30/11/12 | M | x | x | x |  | x |  |  |
| 13 | 30/11/12 | A | x | x | x |  | x |  |  |
| 14 | 7/12/12 | N | x | x | x |  | x |  |  |
| 15 | 18/01/13 | N | x | x | x |  | x |  |  |
| 16 | 25/01/13 | N | x |  | x |  | x |  | x |
| 17 | 28/01/13 | M | x |  | x | x |  |  | x |

**Descriptive Analyses:**

*Scratching:*


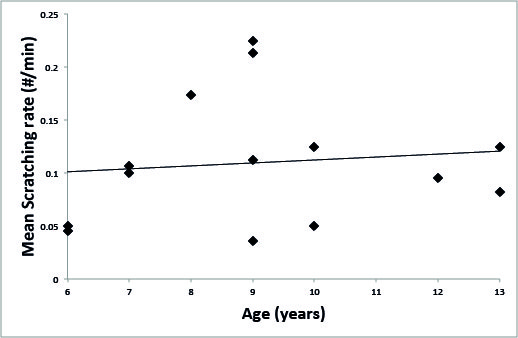


**Figure S1.** Relationship between mean scratching rates (#/min) and age (years), *N* = 14.


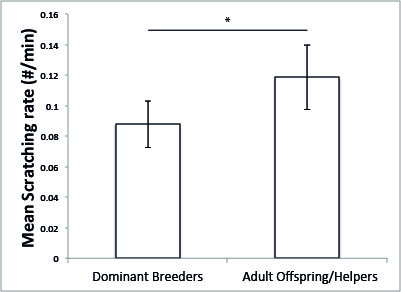


**Figure S2.** Mean ± SEM scratching rates (#/min) of dominant breeders (*N* = 4) and adult offspring / helpers (*N* = 10). * *P* < 0.05.

*
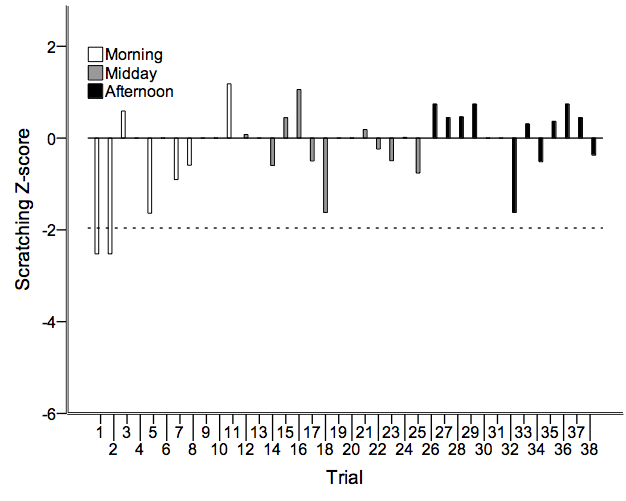
*

**Figure S3.** The distribution of Z-scores from the runs test analyses across all morning, midday and afternoon testing sessions. The dotted line indicates the threshold for significant temporal clumping within a session.

*Stretching*

*
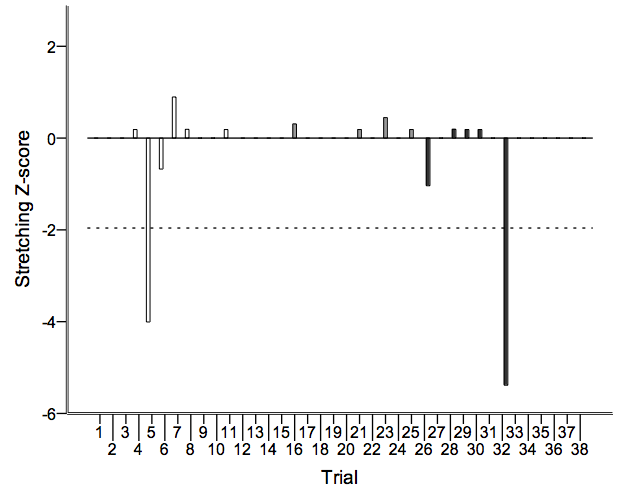
*

**Figure S4.** The distribution of Z-scores from the runs test analyses across all morning, midday and afternoon testing sessions. The dotted line indicates the threshold for significant temporal clumping within a session.

*Tongue protrusion*


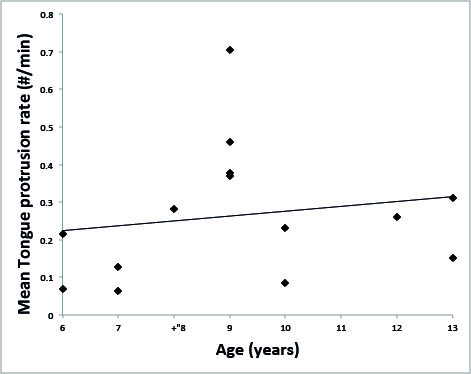


**Figure S5.** Relationship between mean tongue protrusion rates (#/min) and age (years), *N* = 14.


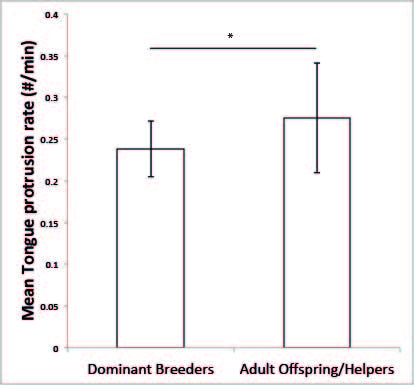


**Figure S6.** Mean ± SEM tongue protrusion rates (#/min) of dominant breeders (*N* = 4) and adult offspring / helpers (*N*= 10). * *P* < 0.05.


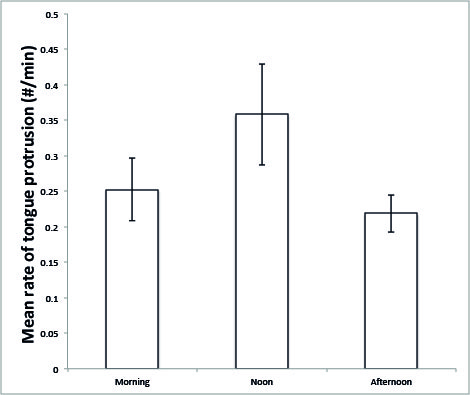


**Figure S7.** Mean ± SEM tongue protrusion rates (#/min) during the morning (*N* = 11), around noon (*N* = 14), and in the afternoon (*N* = 13).


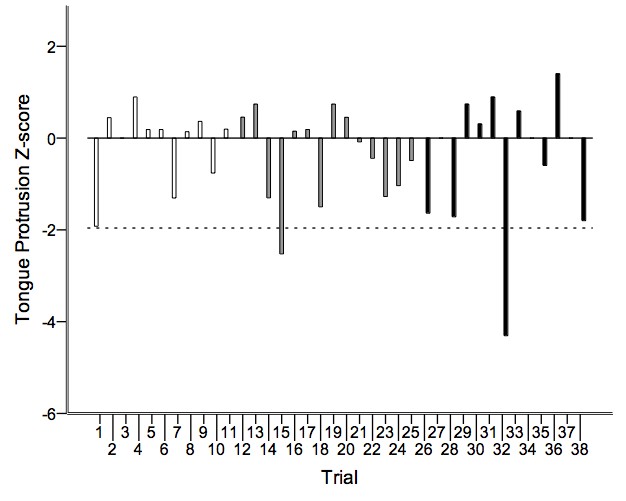


**Figure S8.** The distribution of Z-scores from the runs test analyses across all morning, midday and afternoon testing sessions. The dotted line indicates the threshold for significant temporal clumping within a session.

*Gnawing*


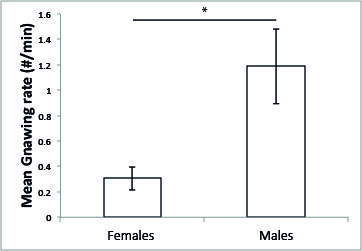


**Figure S9.** Mean ± SEM gnawing rates (#/min) of females (*N* = 7) and males (*N*= 7). * *P* < 0.05.

*Scent-marking*


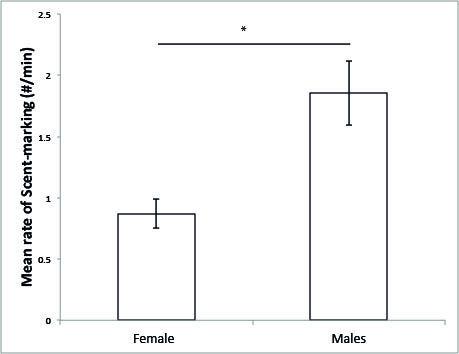


**Figure S10.** Mean ± SEM scent-marking rates (#/min) of females (*N* = 7) and males (*N* = 7). * *P* < 0.05.


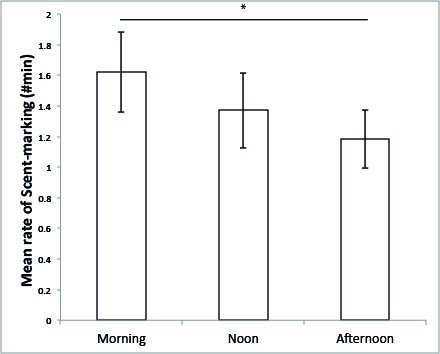


**Figure S11.** Mean ± SEM scent-marking rates (#/min) during the morning (*N* = 11), around noon (*N* = 14), and in the afternoon (*N* = 13). * *P* = 0.05.


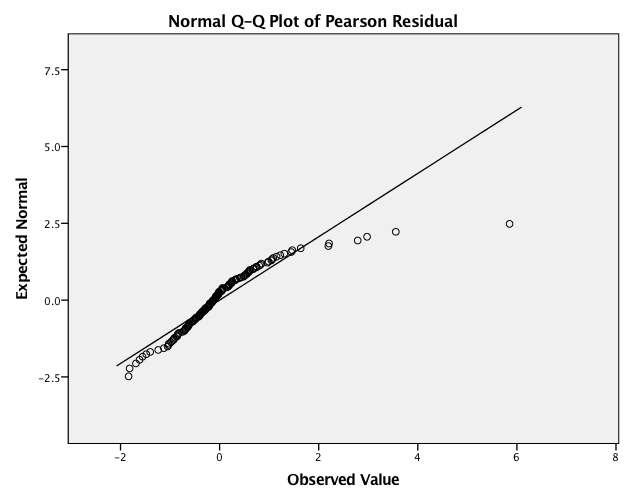


**Figure S12**. Quantile-Quantile (Q-Q) plot comparing the distribution of the residuals of the best fitting model on **Scratching** with a normal distribution.


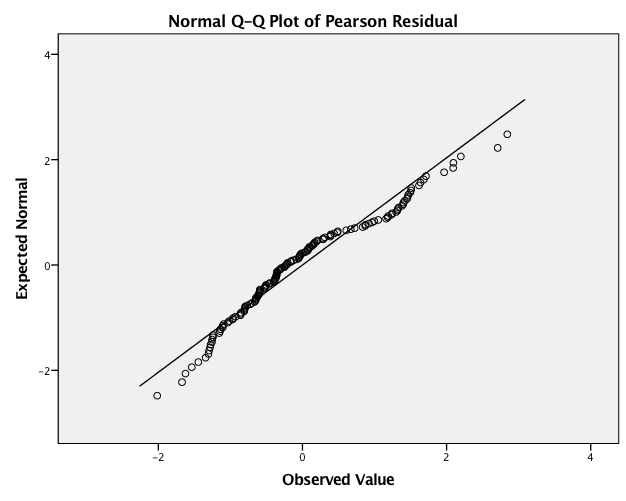


**Figure S12**. Quantile-Quantile (Q-Q) plot comparing the distribution of the residuals of the best fitting model on **Tongue protrusion** with a normal distribution.


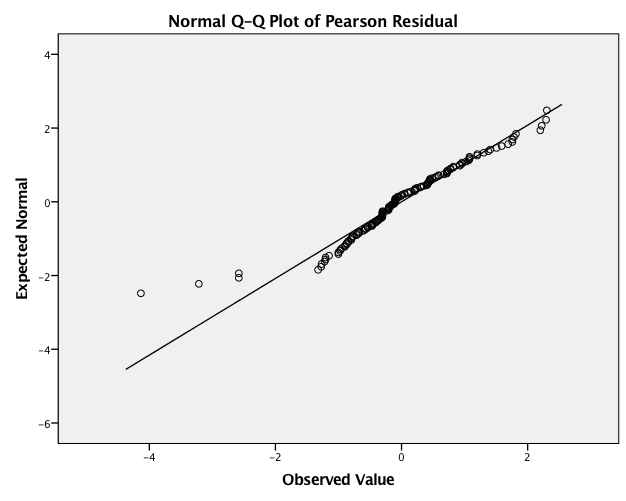


**Figure S13**. Quantile-Quantile (Q-Q) plot comparing the distribution of the residuals of the best fitting model on **Gnawing** with a normal distribution.


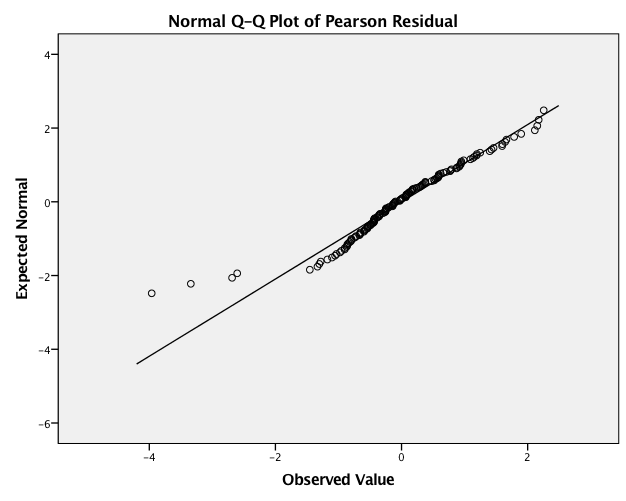


**Figure S14**. Quantile-Quantile (Q-Q) plot comparing the distribution of the residuals of the best fitting model on **Scent-marking** with a normal distribution.
